# A targeted metabolic analysis of football players and its association to player load: Comparison between women and men profiles

**DOI:** 10.3389/fphys.2022.923608

**Published:** 2022-09-30

**Authors:** Gil Rodas, Eva Ferrer, Xavier Reche, Juan Daniel Sanjuan-Herráez, Alan McCall, Guillermo Quintás

**Affiliations:** ^1^ Medical and Performance Department, Barça Innovation Hub, Futbol Club Barcelona, Barcelona, Spain; ^2^ Sports and Exercise Medicine Unit, Hospital Clinic and Sant Joan de Deu, Barcelona, Spain; ^3^ Leitat Technological Center, Terrassa, Spain; ^4^ School of Applied Sciences, Edinburgh Napier University, Edinburgh, United Kingdom

**Keywords:** women sports, training, metabolomics, EPTS, load analysis

## Abstract

Professional athletes undertake a variety of training programs to enhance their physical performance, technical-tactical skills, while protecting their health and well-being. Regular exercise induces widespread changes in the whole body in an extremely complex network of signaling, and evidence indicates that phenotypical sex differences influence the physiological adaptations to player load of professional athletes. Despite that there remains an underrepresentation of women in clinical studies in sports, including football. The objectives of this study were twofold: to study the association between the external load (EPTS) and urinary metabolites as a surrogate of the adaptation to training, and to assess the effect of sex on the physiological adaptations to player load in professional football players. Targeted metabolic analysis of aminoacids, and tryptophan and phenylalanine metabolites detected progressive changes in the urinary metabolome associated with the external training load in men and women’s football teams. Overrepresentation analysis and multivariate analysis of metabolic data showed significant differences of the effect of training on the metabolic profiles in the men and women teams analyzed. Collectively, our results demonstrate that the development of metabolic models of adaptation in professional football players can benefit from the separate analysis of women and men teams, providing more accurate insights into how adaptation to the external load is related to changes in the metabolic phenotypes. Furthermore, results support the use of metabolomics to understand changes in specific metabolic pathways provoked by the training process.

## Introduction

Professional athletes, including football players, undertake a variety of training programs with varying stimuli to achieve desired physiological and psychological adaptations and ultimately enhance their physical performance and technical skills while protecting their health and well-being. A key component of the training process is the daily management and manipulation of the external training load (e.g., minutes/hours, running distances, running speeds through GPS) typically in response to internal loads experienced (e.g., heart rate, rating of perceived exertion) and the subsequent internal responses (e.g., subjective wellness, muscle force production, heart rate recovery, blood lactate concentration, oxygen consumption, etc.). While measuring external training load is common and highly popular, in particular *via* electronic performance tracking systems (Akenhead and Nassis, 2016) (EPTS), as alluded to above, EPTS does not reflect the internal training loads or responses experienced by players. Thus, internal measures are a cornerstone to future exercise prescription, which in the absence of, we cannot be certain how training sessions are affecting players i.e., how they are adapting to, and recovering from sessions and matches (Bourdon et al., 2017) (Arcos et al., 2017). Different internal measures have been described including the rating of perceived exertion (RPE), session RPE (sRPE) (Foster et al., 2001), TRIMP and several modifications to the TRIMP measure, each one of them with limited validity (Passfield et al., 2022). Exercise induces widespread changes in the whole body in a complex network of signaling caused by or as a response to the increased metabolic activity of contracting skeletal muscles (Hawley et al., 2014). Adaptation to these changes leads to genomic, proteomic, and metabolic systemic changes and their integrative analysis has been suggested to provide a more comprehensive description of the effect of exercise than the separate analysis of each level (Impellizzeri et al., 2019) (Hawley et al., 2014). Metabolomics is an area of systems biology that has been gaining traction in sports physiology over the last years. It involves the downstream products of gene regulation and expression, as well as the interaction of a biological system with the environment, providing a meaningful dynamic snapshot of its functional level.

Findings to date in metabolomics sports research have shown short- and long-term exercise-induced changes in metabolic pathways including the amino acids and ATP metabolisms, glycolysis, beta-oxidation of free fatty acids and ketone bodies, and the upregulation of different antioxidant systems (Finaud et al., 2006), (Gorostiaga et al., 2012), (Pechlivanis et al., 2015), (Duft et al., 2017), (Heaney et al., 2017), (Manaf et al., 2018). Analysis of short-term changes after intensive exercise has been also linked with muscle bioenergetics related to the ATP-phosphocreatine (ATP-PCr), and glycolytic systems, and the activation of purine catabolism and lactic acid generation (Enea et al., 2010). Changes in training protocols had an impact on metabolites related to ATP-PCr and lactate metabolisms, purine, fatty acid and branched-chain amino acid (BCAA) degradation, glutamate and Krebs cycle metabolisms, tryptophan and phenylalanine catabolism, oxidative stress, and muscle protein breakdown (Pechlivanis et al., 2010). The analysis of the adaptation of trained cyclists with two endurance-training periods differing in intensity distribution showed changes as well in the metabolic profile specific to each training program, which was attributed to different levels of cellular metabolic–energetic stress experienced. Greater stress was not associated with greater adaptation, suggesting the use of metabolomics to identify novel biomarkers of training stress, adaptation, and recovery (Neal et al., 2013). Focusing on football, short-term changes in the metabolic profiles of young male professional players’ responses revealed a significant post-exercise increase in acylcarnitines involved in fatty acid oxidation, and slight increases in purine metabolites, especially hypoxanthine and xanthine (Alzharani et al., 2020). Another study analyzing metabolic mechanisms in male teenage football players during exercise-induced fatigue reported changes in five metabolic pathways (glycine-serine-threonine metabolism, citrate cycle, tyrosine metabolism, nitrogen metabolism, and glycerophospholipid metabolism) (Cao et al., 2020). The analysis of urine metabolic profiles and EPTS data from 80 professional football male players collected in an observational longitudinal study identified changes in steroid hormone metabolites, hypoxanthine metabolites, amino acids, intermediates in phenylalanine metabolism, tyrosine, tryptophan metabolites, and riboflavin associated with the external load indicating an alteration of biochemical pathways linked to the long-term adaptation to training (Quintas et al., 2020). A recent study assessed urinary metabolomic changes by NMR in elite Brazilian U-20 players in samples collected immediately and 20 h after two soccer matches. Results obtained showed different metabolic profile between athletes with higher and lower RPE values. Athletes with higher RPE values showed a high metabolite profile related to muscle damage (e.g., creatine, creatinine, and glycine) and energy production (e.g., creatine, formate, pyruvate, 1,3 dihydroxyacetone) 20 h post-soccer match (Marinho et al., 2022). Collectively, results indicate that metabolomics can detect changes linked to the physiological adaptation to external load, providing information about fatigue, physical capacity, and performance potentially useful in competitive sports.

The influence of phenotypical sex differences throughout many physiological systems (e.g., respiratory, circulatory and hormonal systems, skeletal muscle) in the integrative metabolic thresholds during exercise has been multiply reviewed (Ansdell et al., 2020), and it is also accepted that sex is among the most relevant biological variables influencing metabolomic and lipidomic profiles (Audano et al., 2018). Despite that, there is a clear underrepresentation of women in clinical studies in sports metabolomics. In a recent systematic review on sports metabolomic studies between 2000 and 2020 (Khoramipour et al., 2021), including football, among the 89 human studies reviewed, 55 (62%) lack of women representation, and only 6 of them (7%) were exclusively targeted to women physiology. So, clearly there is a compelling social need to conduct scientific studies, including in metabolomics, leading to an understanding of the physiological characteristics of female football players and their acute and chronic physiological responses to exercise.

In this context, the objectives of this study were two-fold: to study the correlation between the external load (EPTS) and urinary metabolites as a surrogate of the adaptation to training using uni- and multivariate linear models, and to assess the effect of sex on the physiological adaptations to player load in professional football players by comparing models build using data collected from a male and a female team.

## Materials and methods

**FIGURE 1:**
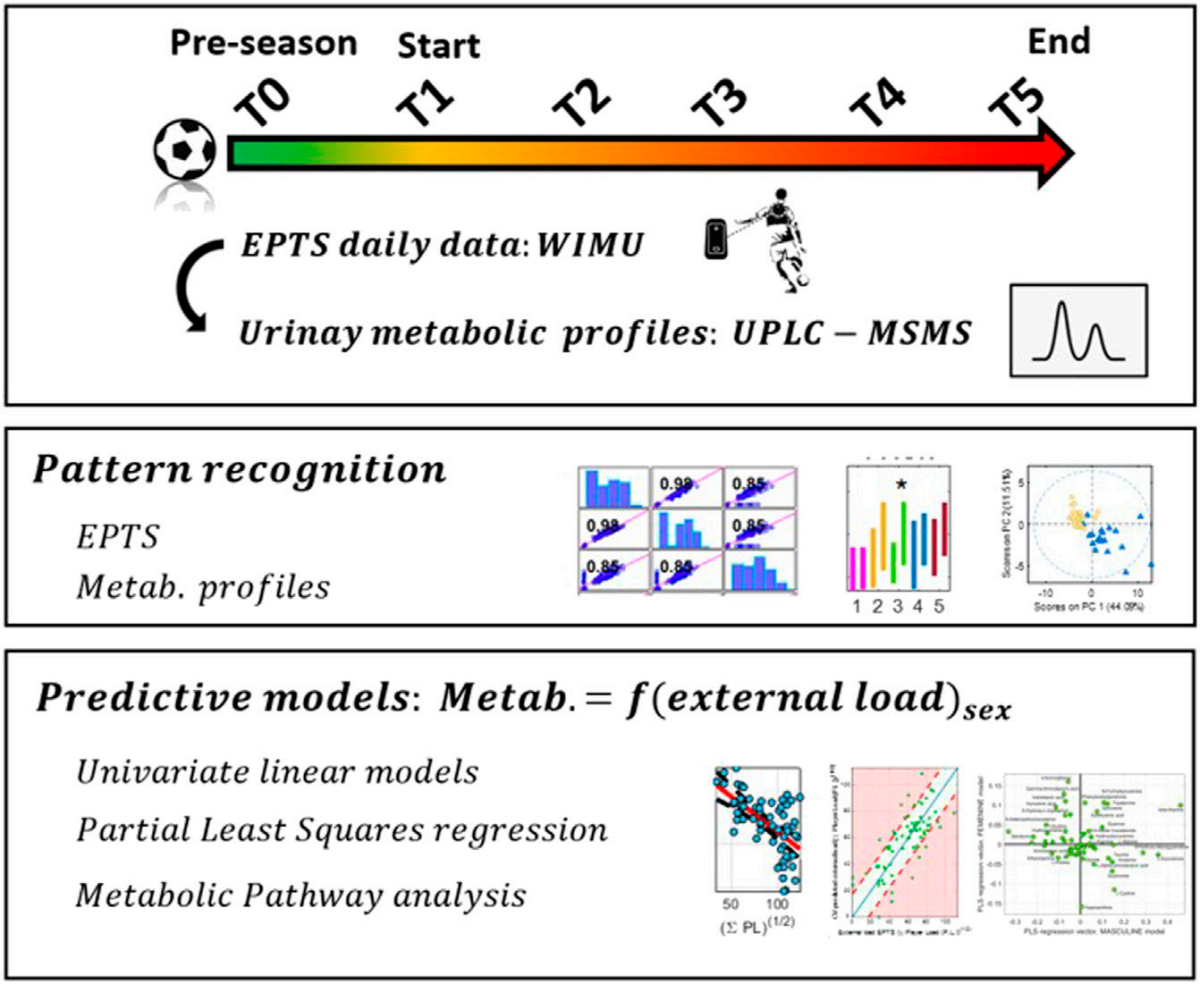
Scheme of the study.

This was a prospective observational, longitudinal study carried out by Futbol Club Barcelona (FCB, Barcelona, Spain) following relevant guidelines and regulations. Institutional board approval for the study was obtained from the Ethics Commission of the Consell Català de l’Esport (Code 012/CEICEGC/2021, Generalitat de Catalunya, Barcelona, Spain). Written informed consent was collected and all data were anonymized to ensure confidentiality. All procedures involving human participants were in accordance with the ethical standards of the institutional and/or national research committee and with the 1964 Helsinki declaration and its later amendments or comparable ethical standards. This study involved the collection of daily EPTS data and urine samples at five time points throughout a season from professional football players of two teams (male and female) (see Figure 1).

### Sample and electronic performance tracking systems data collection

Urine samples and available WIMU PRO™ EPTS data were collected between July 2020 and May 2021. The study included data from 28 professional football players from the women’s FCB team [5 goalkeepers, 8 defenders, 6 midfielders and 9 forwards, age: 25 ± 5 (range 17–36, median: 25) years], and from 23 professional football players from the men’s FCB team [4 goalkeepers, 6 defenders, 3 midfielders and 10 forwards, age: 25 ± 5 (range 18–33, median: 24) years]. Samples from the female team were collected at pre-season following the post-season rest period (t_F1_, *n* = 26), and after 5 (t_F2_, *n* = 23), 7 (t_F3_, *n* = 22), 9 (t_F4_, *n* = 22) and 10 (t_F5_, *n* = 23) months of competition. Samples from the men’s team were collected after 2 (t_M1_, *n* = 20), 8 (t_M2_, *n* = 14), 9 (t_M3_, *n* = 17), 10 (t_M4_, *n* = 13), and 11 (t_M5_, *n* = 10) months of competition. A higher sample collection frequency at the end of the season was included to analyze the effect of a higher physical and psychological stress. Nineteen female players provided samples at all five time points; 4 players at four time points, and 5 players provided samples a single time. Five male players provided samples at all 5 time points; six players at 4 time points, five players at 3 time points, three players at 2 time points, and four players provided a single sample. First morning urine samples were collected to minimize potential confounding from uncontrolled dietary intake, after a day off (rest day) and all the players were advised to avoid strenuous physical activity during the rest day. Urine samples were collected, aliquoted and stored at −80°C until analysis which was carried out within 2 weeks to avoid potential effects of long-term storage.

A description of the training routine using external training load metrics can be found in (Guitart et al., 2022). EPTS daily records included the following variables: absolute High Speed Running (HSR) distance (m), absolute HSR/min (m/min), number of accelerations (+3 m/s^2^)/min (count/min), number of accelerations (+3 m/s^2^)Distance (m), number of accelerations Acc(+3 m/s^2^) (count), number of decelerations (+3 m/s^2^)/min (count/min), number of decelerations (+3 m/s^2^)Distance (m), number of decelerations (+3 m/s^2^) (count), distance (m), distance/min (m/min), duration (min), High Metabolic Load Distance (HMLD) (m), HMLD/min (m/min), Player Load (PL) (a.u.), PL/min (a.u./min), Relative HSR (m), and Relative HSR/min (m/min). Total train time was estimated as the total time (h) from the start to end, pauses included. Distance was defined as the total distance covered (m) including walking, jogging, HSR, and sprinting. HSR distance was the total distance covered at a speed >21 km/h. The HMLD was defined as the distance covered by a player when his metabolic power was abode 25.5 W/Kg. The PL was estimated as:

$$PL_{n}=0.1\sqrt{{\left(X_{n}-X_{n-1}\right)}^{2}+{\left(Y_{n}-Y_{n-1}\right)}^{2}+{\left(Z_{n}-Z_{n-1}\right)}^{2}}$$

and

$$PL=\overset{m}{\underset{n=0}{\sum }}PL_{n}z0.01$$

Where *n* is the order index over time, PL_n_ is the player load calculated at time *n* (i.e., instant player load), X_n_, Y_n_, and Z_n_ are the values of bodyX, bodyY and bodyZ at time *n*. A detailed description of the variables can be found elsewhere (Reche-Soto, 2019; Guitart et al., 2022). EPTS daily records were used to the calculation of cumulative train loads throughout the season, and the total train load since the beginning of the season up to the training day before the sample collection was used for the study of the associations between long-term adaptation and the metabolic profile.

### Metabolic analysis of urine samples

Targeted metabolic analysis focused on tryptophan and phenylalanine pathways and amino acids, previously associated to the physiological adaptation to training in football male players (Quintas et al., 2020). Amino acid analysis was carried out by UPLC-MS/MS following a derivatization step (AccQTag Ultra Derivatization, Waters). The method enabled the quantification of Arginine, Anserine, Methylhistidine, Histidine, Asparagine, Carnosine, Hydroxyproline, Phosphoethanolamine, Serine, Taurine, Aspartic, Citrulline, Ethanolamine, Glutamic acid, *Glycine*, Sarcosine, β-Alanine, Threonine, Hydroxylysine, Glutamine, γ-Aminobutyric acid, Alanine, Lysine, Aminoadipic, β-Aminoisobutyric acid, Proline, Cystine, Cystathionine, Methionine, Ornithine, Tyrosine, α-Aminobutyric acid, Valine, Leucine, Phenylalanine, and Tryptophan. A second analysis focusing on metabolites included in the tryptophan and phenylalanine pathways was carried out by UPLC-MS/MS, and involved the quantification of Aminophenol, Anthranilic acid, 3-Hydroxyanthranilic acid, Tryptamine, Indole-3-acetamide, Phenylalanine, Serotonin, Kynurenic acid, Tryptophan, Xanthurenic acid, Kynurenine, 5-Hydroxy-L-tryptophan, Hydroxykynurenine, N′-Formylkynurenine, Indolelactic acid, p-Tyrosine, Phenylacetylglutamine (PAGN), Guanine, Guanosine, 8-hydroxydeoxyguanosine (8-OHdG), S-Adenosylhomocysteine (SAHC), S-Adenoylmethionine (SAM), and Hypoxanthine. All metabolite concentrations were expressed normalized by their corresponding creatinine urinary concentration. Detailed descriptions of the analytical procedures are included in the Supplementary Material.

### Statistical analysis

To increase the robustness of the results, metabolites were excluded from further analysis if the number of missing values (i.e., concentrations below the lower limit of quantification) was >20%. To avoid redundancy in the data, for those metabolites analyzed by two methods (Tryptophan, Phenylalanine, and Tyrosine) only the concentrations obtained from the aminoacid analysis were used. The final data set comprised concentrations of 56 metabolites. Two samples from the female team and one from the male team were excluded from further analysis based on an initial principal component analysis (PCA) carried out for the identification of outliers. Pairwise Pearson’s correlations and their corresponding *p*-values were calculated for testing the null hypothesis of no correlation between EPTS variables against the right-tailed alternative that the correlations are greater than zero. PCA and partial least squares (PLS) regression were carried out using autoscaled data to adjust for the differences in ranges among metabolites. The selection of the number of latent variables in the PLS models, and the estimation of its generalization performance was based on the root mean square error of cross validation (RMSECV) values obtained using a leave-one player-out cross-validation (CV) approach. Accordingly, during CV, the data set was split into *k* folds where each fold included all samples collected from a single player. The statistical significance of the RMSECV was assessed by permutation testing (*n* = 2000 permutations), as described elsewhere (Rubingh et al., 2006). The importance of each variable in the PLS models was estimated using the Variable Importance in the Projection (VIP) score (Chong and Jun, 2005). Over Representation Analysis (ORA) was carried out using a set of 84 metabolite sets based on the Kyoto Encyclopaedia of Genes and Genomes (KEGG) library of human metabolic pathways, a cut-off *p*-value = 0.05, and a minimum of 3 hits/pathway.

## Results

EPTS data and metabolic profiles were initially analyzed for pattern recognition to identify associations among EPTS variables, and main differences in the EPTS and metabolic profiles between the two teams over the season. Then, separate univariate (linear) and multivariate (PLS) regression models were built for each team to describe the association between the external load and the metabolic profiles. Over Representation Analysis (ORA) was used to examine enriched metabolic pathways associated with the external load to support the biological interpretation of the regression models.

### Data overview

Analysis of the EPTS variables collected throughout the season demonstrated a significant cross-correlation in both teams, as it can be seen in Supplementary Figures S1,S2, where correlation plots among pairs of EPTS variables are depicted for each team separately. The observed high and statistically significant (*p*-value<0.05) correlations across EPTS features in both teams enabled the selection of a single variable (player load) as a surrogate of the external training load, which showed correlations in the 0.90–0.99 (female team) and 0.83–0.98 (men’s team) ranges with the other EPTS variables with the exceptions of Rel. HSR in the female team (see Supplementary Figure S2), and Abs HSR and Rel HSR in the male team (see Supplementary Figure S3). Player load is one of the most frequently used load indicators and it shows the combination of the accelerations produced in the three main anatomical planes, leading to estimation of the total load (Reche-Soto et al., 2019). Moreover, PL has been found strongly correlated with variables like the heart rate and VO_2max_ (Barrett et al., 2016), subjective RPE (Casamichana et al., 2013), as well as high test-retest and inter and intra-device reliability in continuous (Barrett et al., 2016) and intermittent efforts. Changes in the distributions of the urinary concentrations of the metabolites over time in response to training were then analysed. As it can be seen in Supplementary Figure S3, the concentrations of the metabolites across the season showed a high overlap in both teams. Nonetheless, the gender differences were evaluated at each time point using a univariate *t*-test, and a *p*-value < 9 10^−4^ was considered statistically significant. Results found statistically significant differences between the male and female teams in a total of 52 of the 56 metabolites in at least one of the time points.

**FIGURE 2:**
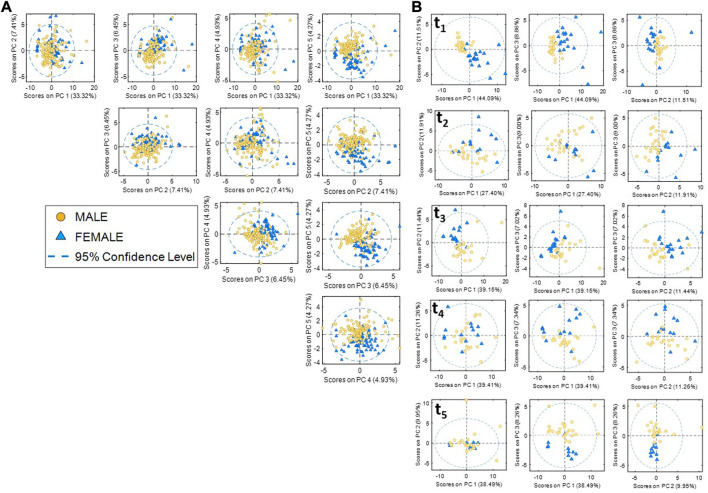
Principal component analysis (PCA) of metabolomic data. **(A)** Scores plots of metabolomic data (male and female data sets). **(B)** Scores plots of metabolic data at each sample collection point.

Then, a multivariate pattern recognition analysis was carried out to consider the interrelation of metabolites and their complementary behavior with the effect of training and sex. Accordingly, PCA was selected to identify the main sources of variation in the metabolic profiles which could be associated with consequences of physical training throughout a season. Scatter plots of PCA scores are shown in Figure 2A for the first five principal components representing 56% of the data variance. Results showed the overlap of the metabolic profiles of men and women in the first four PCs summarizing 52% of the data variance, in agreement with results shown in Supplementary Figure S2. However, results also showed a sex-related cluster along the fifth PC (4.5% variance), indicating that sex was among the main sources of variance in the data set, along with other effects such as the collection time point that may have a larger weight on the metabolic profiles. To further estimate the weight of sex on the data variation, the separate analysis of samples of each team collected at each time point was carried out. The scores plot depicted in Figure 2B revealed a much larger clustering of male and female samples in the first three PCs at every time point.

### Longitudinal analysis of metabolic changes associated to the external load

Initially, a simple univariate linear model approach was used to capture the quantitative associations of the metabolite concentrations with the cumulative training over a season, using the EPTS feature ‘player load’ as a surrogate of the total external load. Results obtained for each metabolite in each team are summarized in Supplementary Figures S4,S5. Results from the analysis of thefemale sample set revealed that 17 metabolites (30% of the total included in the study) showed statistically significant linear regression fits (*p*-value<9 10^−4^ for the t-statistic of the hypothesis test that the corresponding slope coefficient is equal to zero or not): 16 with a positive slope (i.e., higher concentrations at increasing external loads) and 1 (hypoxanthine) with a negative slope (i.e., decreasing concentrations at increasing external loads). Over Representation Analysis (ORA) was used to determine whether known metabolic pathways were over-represented (i.e., enriched) in the experimentally-derived list of metabolites associated with the training load. Using the list of 17 linearly associated metabolites, ORA detected the Tryptophan metabolism (*p*-value = 9 10^−3^) as overrepresented.

Following the same strategy, the analysis of the male sample set revealed that 12 metabolites (21% of the total included in the study) showed statistically significant linear regression fits (*p*-value<9 10^−4^): 3 of them (β-alanine, guanine, and guanosine) with a positive slope (i.e., higher concentrations at increasing external loads) and 9 (citrulline, hydroxy-lysine, glutamine, 3-aminoisobutanoic acid, proline, cystathionine, methionine, ornithine, and SAHC) with a negative slope (i.e., decreasing concentrations at increasing external loads). In this case, ORA detected the Arginine biosynthesis (*p*-value = 10^−4^), the Cysteine and methionine (*p*-value = 2 10^−3^), and Purine (*p*-value = 0.01) metabolisms and the aminoacyl-tRNA biosynthesis (*p*-value = 5 10^−3^) as overrepresented.

**FIGURE 3:**
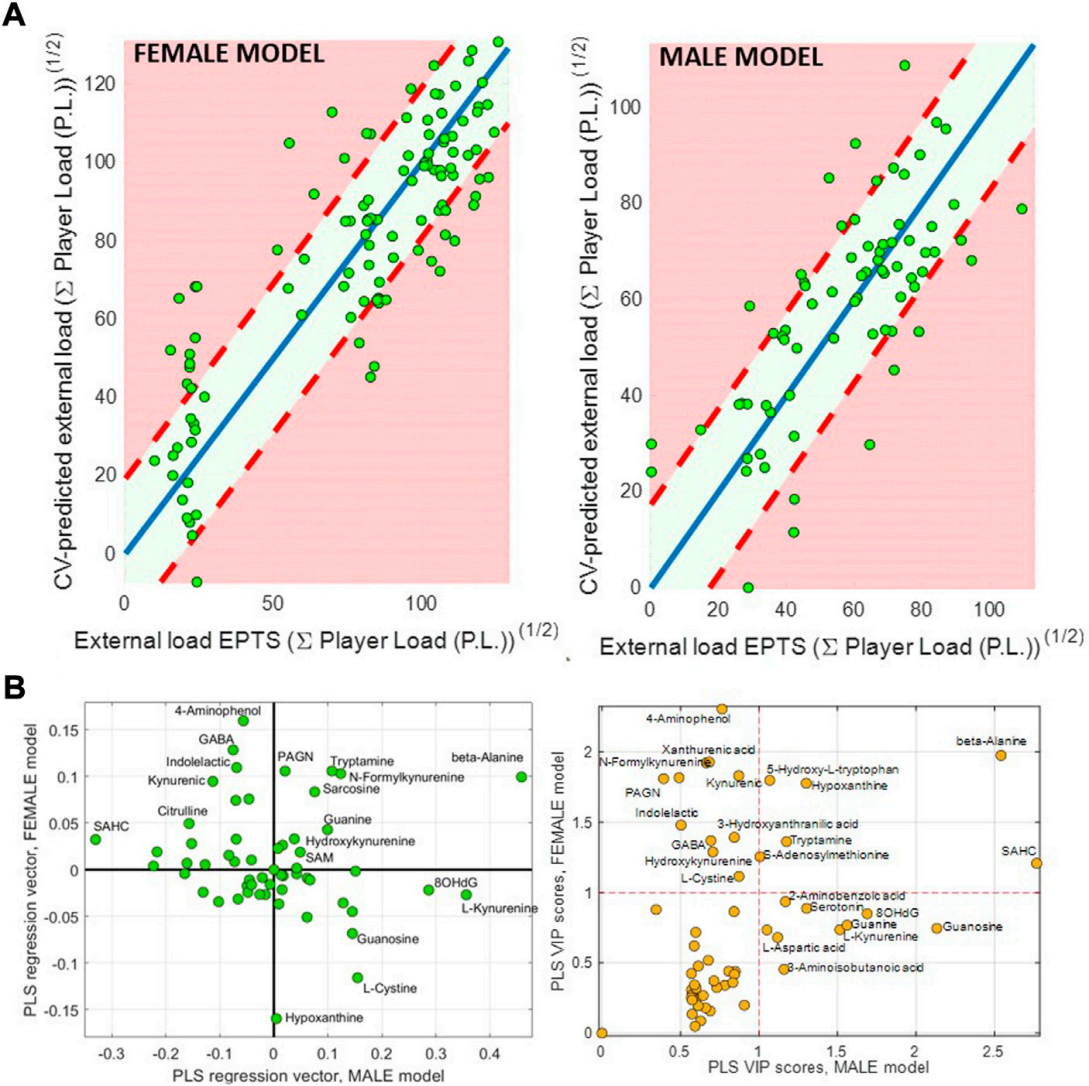
Partial Least Squares (PLS) analysis. **(A)** cross-validation (CV) predicted values in the female and male teams. **(B)** Plot of the PLS regression (left) and VIP score (right) vectors in the PLS models built for the female and male teams.

Multivariate PLS models were built to further investigate the relationship between the urinary metabolic profiles and the external load, and the degree of dissimilarity between the models built for the female and male data sets. Figure 3A shows the cross-validated predicted external load values for the female and male samples, based on their metabolic data. On the one hand, these results provided statistically significant (*p*-value<5 10^−3^) RMSECV values in both models, supporting the hypothesis of quantitative shifts in the metabolome associated with physiological adaptation. On the other hand, low comparability between the male and female models was observed, as shown by the low correlation between the PLS regression vectors of the male and female models (see Figure 3B), which might imply different adaptations to training. Differences in the sign of coefficient in the regression vectors agreed with the differences observed in the previous linear models (see e.g., 5-hydroxy-tryptophan, that showed a positive value in the PLS regression vector of the female model and a negative one in the male model). Figure 3B shows the calculated VIP scores for the two PLS models, that also displayed a limited correlation between them.

## Discussion

Metabolic analysis of a list of endogenous compounds that included aminoacids, tryptophan, and phenylalanine metabolites, among other metabolites, enabled the detection of changes in the urinary metabolome linked to the total external load in men’s and women’s team players throughout a football season. This result showing the association between the external load and urinary metabolites could be used as a surrogate of the adaptation to training. Besides, a significant effect of sex on the physiological adaptations to player load could be observed.

The concentration ranges of the metabolites at the 5 collection points shown in Supplementary Figure S2 showed a high overlap across the season in both teams. However, statistically significant differences between men and women were found in a total of 52 of the 56 metabolites in at least one of the time points. Unsupervised PCA of metabolic profiles at each sample collection point revealed the clustering of male and female samples in the first three PCs (see Supplementary Figure S2). Although gender-specific metabolic differences might originate from either biological or socially influenced gender effects (Krumsiek et al., 2015), these results confirmed that gender has a significant impact in the baseline concentrations in studies of association between external load and metabolic profiles.

Univariate linear models to test the association between the metabolite concentrations and the external loads showed a significant impact of training on the metabolome, with 21% and 30% of the metabolites showing statistically significant linear regression fits in in the men and women’s team, respectively. However, only two metabolites were linearly associated with the cumulative external load in both men and women: beta-Alanine (positive association in both teams) and SAHC (negative and positive association in the female and male team, respectively) (see Supplementary Figures S4,S5), suggesting significant differences of the effect of training on the metabolic profiles. Results from the set of univariate models were further used for pathway analysis to support the identification of associations between pathways linked to the adaptation to training. The basic hypothesis of ORA in this context is that relevant pathways can be detected if the proportion of differential expressed metabolites, within a given pathway, exceeds the proportion of metabolites that could be randomly expected (García-Campos et al., 2015), using Fisher’s exact test to test the null hypothesis of no association between the compounds in the pathway and the outcome of interest (Xia and Wishart, 2010; Wieder et al., 2021). ORA detected the Tryptophan metabolic pathways as overrepresented in the female team. In the male team, on the other hand, ORA detected the Arginine biosynthesis, the Cysteine and methionine, and Purine metabolisms as well as the aminoacyl-tRNA biosynthesis as overrepresented. The difference in the overrepresented pathways supports the hypothesis of characteristic adaptations to chronic exercise in the men’s and women’s team players.

Furthermore, the statistical significance of the multivariate PLS models confirmed the relationship between the urinary metabolic profiles and the external load in both teams. However, PLS regression coefficients from the two models depicted in Figure 3B indicated low comparability between them, which would imply different adaptations. Results revealed also significant differences between the top ranked metabolites in the two models using the Variable Importance in the Projection (VIP) scores. In the female model, several tryptophan metabolites including aminophenol, kynurenic acid, xanthurenic acid, 5-hydroxy-tryptophan, N-formylkynurenine, indolelactic acid, hydroxy-kynurenine, tryptamine, and hydroxy-anthranilic were among the top-VIP ranked metabolites, suggesting a progressive change in the tryptophan metabolism. Exhaustive aerobic exercise has also been associated with increased immune activation and alterations in monoamine metabolism in trained athletes (Strasser et al., 2016). Besides, β-Alanine, GABA, sarcosine and hypoxanthine were among the top-ranked metabolites in the multivariate model. β-Alanine, is a rate-limiting factor to the intramuscular synthesis of carnosine and, although the results reported are controversial, its supplementation has been associated with improvements in exercise performance (Lancha Junior et al., 2015). β-Alanine urinary concentrations were associated to larger player loads over the season in both teams. Increased urinary concentrations of β-alanine can be linked to a higher breakdown of the pyrimidine bases cytosine and uracil. Higher concentrations of β-Alanine could also indicate altered carnosine homeostasis. β-Alanine is also formed *in vivo* by the degradation of carnosine, a dipeptide consisting of the amino acids β-Alanine and histidine, and increases in muscle carnosine content have been hypothesized to be an adaptation to long-term high-intensity training (Perim et al., 2019). GABA has a role as an inhibitory neurotransmitter in the central nervous system and participates in the physiologic adjustment of pituitary gland function and control of the growth hormone secretion from the pituitary gland (Godfrey et al., 2003; Sakashita et al., 2019), which plays a key role in skeletal muscle growth and maintenance, in the amino acid transport and in the insulin growth factor-1 production (GF1), which in turn promotes muscle protein synthesis. It has been previously reported that purine metabolism reflects the exercise-induced muscle adaptations and training status of highly trained athletes (Zieliński and Kusy, 2015). As hypoxanthine is related to purine degradation, lower resting urinary hypoxanthine levels may indicate a training-induced adaptation in purine nucleotide metabolism (Kistner et al., 2019).

The VIP scores of the male model included SAHC, guanosine, guanine, hypoxanthine, and 8-OHdG among the top ranked metabolites. 8-OHdG is one of the predominant forms of free radical-induced oxidative lesions, and it is widely used as a biomarker for oxidative stress. The observed slight increase in the urinary concentrations of this oxidatively generated nucleic acid modification could suggest an insufficient antioxidative adaptation following training programs (Larsen et al., 2020). The minor increase in hypoxanthine urinary concentrations as a function of the total training load in the men’s team could indicate a higher purine nucleotide degradation or less efficient hypoxanthine salvage process, compared to the negative association of hypoxanthine and training load observed in the female model. Previous results have shown that intermittent sprint training reduces the total urinary purine excretion (Stathis et al., 2006). The increase in the concentrations of guanine, a derivative of purine, could also be the consequence of an adaptation in the purine metabolism.

Developing objective strategies to monitor adaptation to training and eventually, for the early detection of an impaired recovery or adaptation, is critical to strength the interaction between trainers and athletes to improve personalize training in collective sports and prevent muscle injuries. Collectively, our results demonstrate that the development of metabolic models of adaptation in professional football players can benefit from the separate analysis of female and male teams, providing more accurate insights into how the external load is related to changes in the metabolic phenotypes. However, to define clear criteria to classify players into different adaptation profiles, further analysis of longitudinal changes in the metabolomic profiles of larger populations (e.g., different teams and clubs) and the impact of experimental factors (e.g., sampling frequency), as well as the comparison of different metrics for the assessment of adaptative responses to training loads should be carried out. Moreover, future research will need to assess the impact of variables not considered in this study such as menstrual cycle disturbances, use of contraceptives, additional recreational lifestyle activities, and diet also linked to the energy availability and to the concept of RED-S (Relative Energy Deficiency in Sports) (Mountjoy et al., 2018), which in women should be paid special attention.

## Conclusion

The development of new measures of internal load to monitor the adaptation to exercise of elite players is an active field that could pave the way to more effective personalized training strategies and facilitate the study of the association of the external and internal loads with outcomes such as the incidence of muscle injuries or physical performance. In this context, sex is a frequently overlooked critical factor to consider. Here, targeted metabolic analysis of a set of endogenous compounds including aminoacids, and tryptophan and phenylalanine metabolites enabled the detection of changes in the urinary metabolome associated with the external training load throughout a complete season in professional male and female football teams. However, univariate and multivariate regression analysis, as well as ORA, showed significant differences in the changes observed in the male and female teams, mainly linked to the Tryptophan, Cysteine and methionine metabolisms, Purine metabolism, and Arginine and aminoacyl-tRNA biosynthesis. Collectively, our results demonstrate that the development of metabolic models of adaptation in professional football players can benefit from the separate analysis of women and men teams, providing more accurate insights into how the external load is related to changes in the metabolic phenotypes. Furthermore, results support the use of metabolomics to understand changes in specific metabolic pathways provoked by the training process.

## Data Availability

The original contributions presented in the study are included in the article/Supplementary Materials, further inquiries can be directed to the corresponding author.
