# Supplementary material for: A targeted metabolic analysis of football players and its association to player load: Comparison between women and men profiles
Source: Front Physiol. 2022 Sep 30;13:923608. doi: 10.3389/fphys.2022.923608 (PMC9561103; doi:10.3389/fphys.2022.923608)

Supplementary Material

Metabolic analysis of urine samples

Targeted metabolic analysis focused on tryptophan pathway and amino acids profiles, previously associated to the physiological adaptation to training in football players[1].

Quantification of Tryptophan metabolic pathway

Urine samples were thawed at room temperature on ice, homogenized (vortex, 10 s) and centrifuged at 10,000 *g* (4 °C, 10 min). Then, 100 µl of supernatant were retained and 300 µl of H_2O (0.1 % v/v HCCOH) were added. Samples were homogenized (vortex, 10 s) and centrifuged at 10,000 *g* (4 ° C, 10 min). A total of 100 µL of supernatant were transferred to a 96-well plate where each sample was spiked with 5 µl of an internal standard solution. UPLC-MS analysis was carried out as described elsewhere[2] with minor modifications. The internal standard solution included hydroxytryptophan-D_4, L-kynurenine-D_4, indole-D_5-3-acetamide, 4-chloro-kynurenine-$^{13}C_2$, ^{15}N, 6-hydroxymelatonin-D_4, kynurenic acid-D_5, PAGN-D_5, phenylalanine-D_5, serotonin-D_4, tryptamine-D_4, tryptophan-D^5, xanthurenic acid-D_4 and phenylalanine-D_5 (1000 nM each) in CH_3CN:H_2O (2:98) (0.1% v/v HCOOH). Samples were analysed using an Acquity HSS T3 C18 (100 x 2.1 mm, 1.8 µm) column. Mobile phases were H_2O (0.1% v/v HCOOH) (A) and (0.1% v/v HCOOH) CH_3CN (B). Gradient elution was phase B held at 2% from 0 to 0.5 min; it then increased linearly to 45% over the next 5 min. Then, phase B was increased to 90% in 0.2 min followed by a fast return to initial conditions between 5.7 and 6 min, which were held for 1.5 min for column re-equilibration. Injection volume, flow rate and column temperature were set at 3 mL, 550 mL/min and 55 ℃, respectively. The autosampler temperature was 6 ℃. Electrospray ionisation conditions were capillary 2.9 kV, cone 25 V, source temperature 120 ℃, and desolvation temperature 395 ℃; N_2 cone and desolvation gas flow rates were 150 and 800 L/ h, respectively.

The targeted analysis included the following metabolites: Aminophenol (HMDB0001169), Anthranilic acid (HMDB0001123), 3-Hydroxyanthranilic acid (HMDB0001476), Tryptamine (HMDB0000303), Indole-3-acetamide (HMDB0029739), Phenylalanine (HMDB0000159), Serotonin (HMDB0000259), Kynurenic acid (HMDB0000715), Tryptophan (HMDB0000929), Xanthurenic acid (HMDB0000881), Kynurenine (HMDB0000684), 5-Hydroxy-L-tryptophan (HMDB0000472), Hydroxykynurenine

(HMDB0000732), N'-Formylkynurenine (HMDB0001200), Indolelactic acid (HMDB0000671), p-Tyrosine (HMDB0000158), Phenylacetylglutamine (HMDB0006344), Guanine (HMDB0000132), Guanosine (HMDB0000133), 8-hydroxydeoxyguanosine (8-OHdG, HMDB0003333), S-Adenosylhomocysteine (SAHC, HMDB0000939), S-Adenoylmethionine (SAM, HMDB0001185), and Hypoxanthine (HMDB0000157).

Quantification of aminoacids

Amino acid analysis was carried out following a derivatization step before their analysis by UPLC-MS/MS. AccQTag Ultra Derivatization kit (Waters) was used according to manufacturer's protocol, as described below. Urine samples were thawed on ice, homogenized on vortex during 10 s and centrifuged at 10,000 g (4 °C, 10 min). Then, 70 µL of water (Optima LC-MS grade, Fisher Scientific) were added to 5 µL of urine sample and were vortexed 10 s. 10 µL of supernatant were mixed with 70 µL of AccQTag Ultra borate buffer and 10 µL of 1 µM IS solution mix. Then, 20 µL of AccQ•Tag reagent (6-aminoquinolyl-N-hydroxysuccinimidyl carbamate in acetonitrile) were added, and the reaction was allowed to proceed for 10 min at 55 °C. Finally, samples were transferred to a 96 well-plate and analysed by ULPC-MS/MS. Quantitative analysis of amino acids was performed employing a 1290 Infinity UPLC system from Agilent equipped with a UPLC CORTECS C18 column (150 × 2.1 mm, 1.6 µm) from Waters coupled to an Agilent 6460 triple quadrupole MS system operating in ESI+ mode. The flow rate was set to 500 µL min^{-1} running a binary gradient with 0.1% v/v HCOOH in H_2O and 0.1% HCOOH in CH3CN. Column and autosampler temperatures were selected at 55 and 4 °C respectively, and injection volume was 3 µL. Electrospray ionization conditions were capillary 4000 V, gas temperature 300̊C, gas flow 10 L/min, nebulizer 45 psi, sheath gas temperature 300̊C, sheath gas flow 12 L/min and nozzle voltage 500 V. The analysis included the following metabolites: Arginine (HMDB0000517), Anserine (HMDB0000194), Methylhistidine (HMDB0000001), Histidine (HMDB0000177), Asparagine (HMDB0000168), Carnosine (HMDB0000033), Hydroxyproline (HMDB0240251), Phosphoethanolamine (HMDB0000224), Serine (HMDB0062263), Taurine (HMDB0000251), Aspartic (HMDB0000191), Citrulline (HMDB0000904), Ethanolamine (HMDB0000149), Glutamic acid (HMDB0000148), Glycine (HMDB0000123), Sarcosine (HMDB0000271), β-Alanine (HMDB0000056), Threonine (HMDB0000167), Hydroxylysine (HMDB0000450), Glutamine (HMDB0000641), γ-Aminobutyric acid (HMDB0000112), Alanine (HMDB0000161), Lysine (HMDB0000182), Aminoadipic (HMDB0000510), β-Aminoisobutyric acid (HMDB0003911), Proline (HMDB0000162), Cystine (HMDB0000192), Cystathionine

(HMDB0000099), Methionine (HMDB0000696), Ornithine (HMDB0000214), Tyrosine (HMDB0000158), α-Aminobutyric acid (HMDB0000452), Valine (HMDB0000883), Leucine (HMDB0000687), Phenylalanine (HMDB0000159), and Tryptophan (HMDB0000929).

All metabolite concentrations in the samples were expressed normalized by their corresponding creatinine concentration quantified following the protocol of the Urinary Creatinine Detection Kit (Arbor Assays™, Ann Arbor, MI), employing a dilution factor of 1:4 and measuring the absorbance at 490 nm.

References

[1] G. Quintas, X. Reche, J.D. Sanjuan-Herráez, H. Martínez, M. Herrero, X. Valle, M. Masa, G. Rodas, Urine metabolomic analysis for monitoring internal load in professional football players, Metabolomics Off. J. Metabolomic Soc. 16 (2020) 45. https://doi.org/10.1007/s11306-020-01668-0.

[2] S. Lario, M.J. Ramírez-Lázaro, D. Sanjuan-Herráez, A. Brunet-Vega, C. Pericay, L. Gombau, F. Junquera, G. Quintás, X. Calvet, Plasma sample based analysis of gastric cancer progression using targeted metabolomics, Sci. Rep. 7 (2017) 17774. https://doi.org/10.1038/s41598-017-17921-x.

Supplementary Figure 1. Pair-wise correlations among cumulative values of the seventeen considered EPTS variables in the women's (top) and men's (bottom) football teams. Variables: 1: Abs HSR (m), 2: Abs HSR/min (m/min), 3: Acc (+3 m/s²)/min (count/min), 4: Acc +3 m/s²Dist (m), 5: Acc+3 m/s² (count), 6: Dec (+3 m/s²)/min (count/min), 7: Dec +3 m/s² Dist (m), 8: Dec+3 m/s² (count), 9: Dist (m), 10: Dist/min (m/min), 11: Duration (min), 12: HMLD (m), 13: HMLD/min (m/min), 14: PL (a.u.), 15: PL/min (a.u./min), 16: Rel HSR (m), 17: Rel HSR /min (m/min).

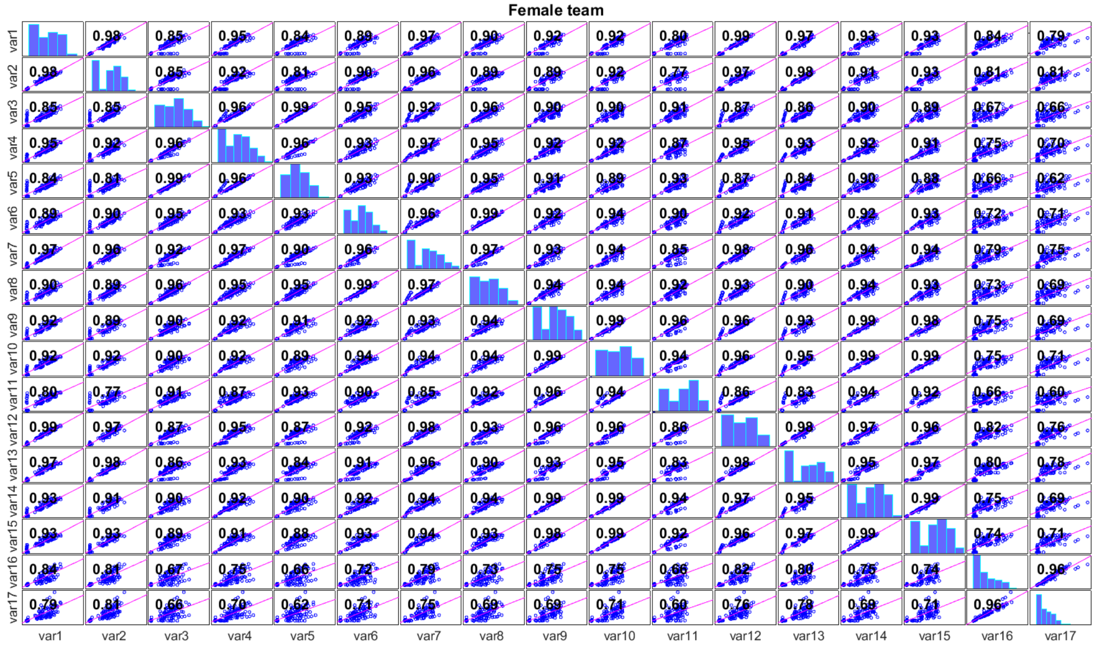

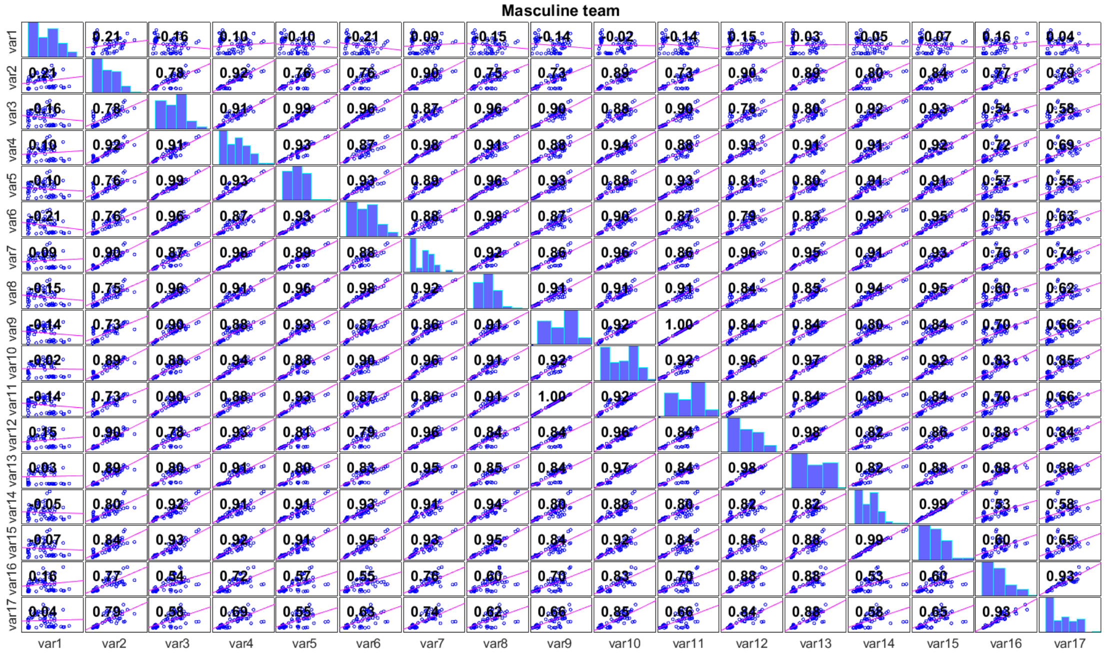

Supplementary Figure 2. Bar plots showing the 5-95% percentiles of the concentrations of each metabolite as a function of the sample collection point. For each time-point, the left and right bars represent the distributions of the female and male teams, respectively. Gender differences were assessed at each time point using a univariate t-test. Statistically significant differences between men and women (t-test p-value<0.05) are indicated by an *.

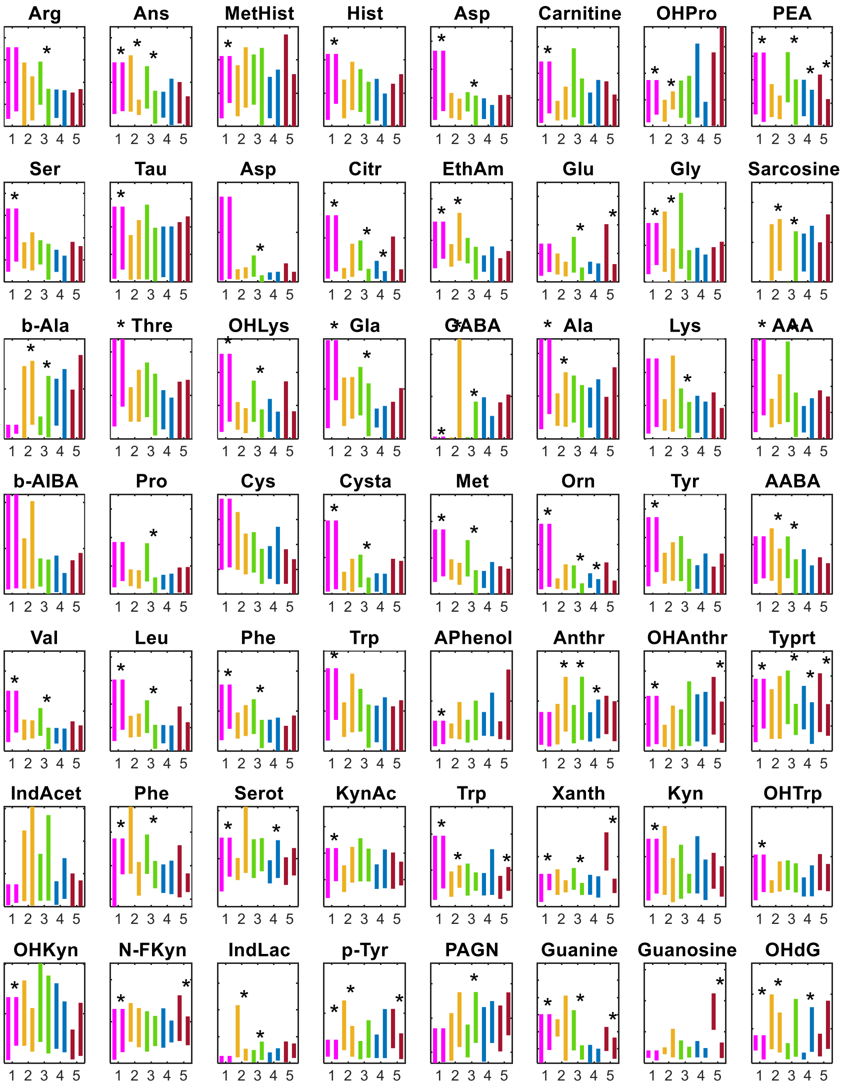

Supplementary Figure 3. Linear models estimated to assess the association between the metabolite log-transformed concentrations and the cumulated Player Loads [AU] in the women's (A) and men's (B) teams. Statistically significant models are highlighted in red.

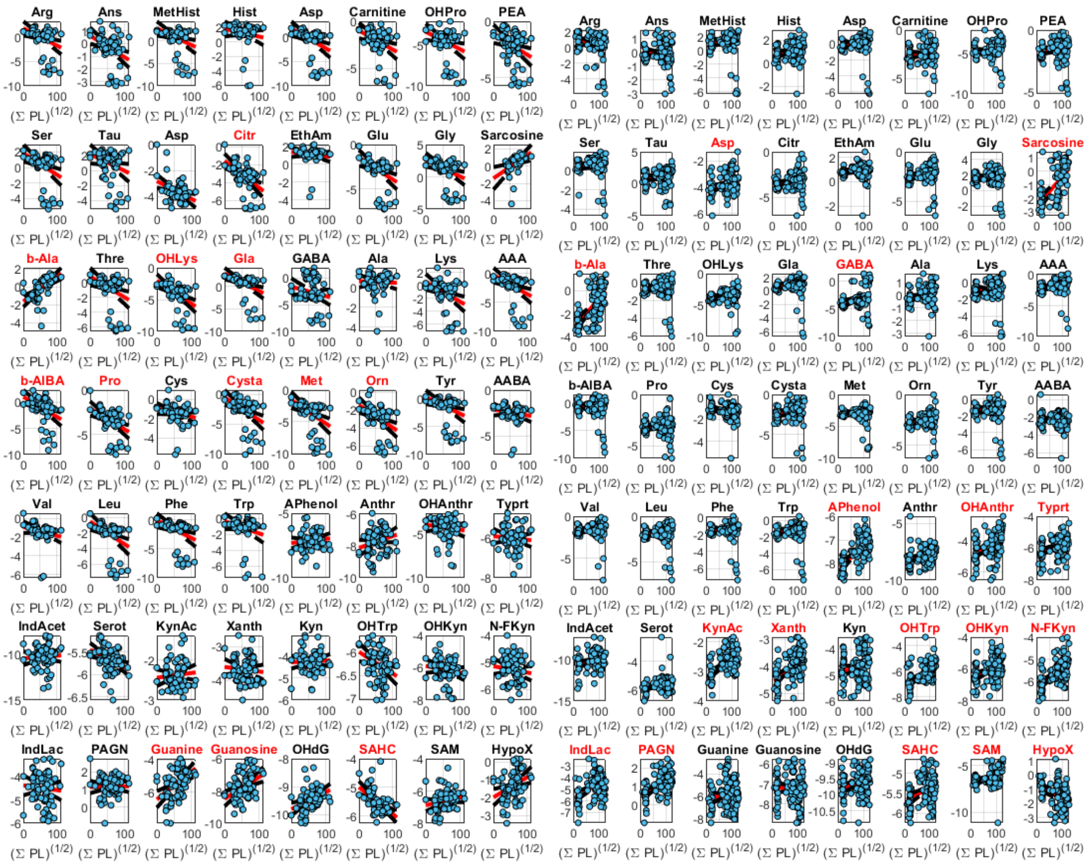

Supplement: Supplementary file 1 [file DataSheet2.PDF]
